# Extreme anorexia nervosa: medical findings, outcomes, and inferences from a retrospective cohort

**DOI:** 10.1186/s40337-020-00303-6

**Published:** 2020-06-23

**Authors:** Dennis Gibson, Ashlie Watters, Jeana Cost, Margherita Mascolo, Philip S. Mehler

**Affiliations:** 1 ACUTE Center for Eating Disorders at Denver Health, 723 Delaware St, Pav M 3rd floor, Denver, CO 80204 USA; 2 Department of Medicine, University of Colorado School of Medicine, 13001 E 17th Pl, Aurora, CO 80045 USA; 3 Eating Recovery Center, 7351 E Lowry Blvd, Denver, CO 80230 USA

**Keywords:** Extreme anorexia nervosa, Medical complications, Malnutrition, Outcomes, Refeeding

## Abstract

**Background:**

Extreme anorexia nervosa (AN) is defined as a BMI < 15 kg/m^2^ in those meeting DSM-V diagnostic criteria for AN. This study seeks to define the frequency of medical complications in this group of patients in order to help inform the care of individuals < 65% ideal body weight who seek treatment for their extreme eating disorders.

**Methods:**

Through retrospective chart review and computerized data collection, we obtained the baseline characteristics and medical findings of 281 adult patients, with AN restricting and binge-purge subtypes, admitted to the ACUTE unit for medical stabilization between May 2013 and August 2018.

**Results:**

In this population, with a mean admitting BMI of 12.1 kg/m^2^ (range = 7.5–15.7), 56% admitted with bradycardia, 45% demonstrated increased liver function tests (LFTs) on admission, 64% admitted with leukopenia, 47% with anemia, and 20% presented with thrombocytopenia. During admission, 38% developed hypoglycemia, 35% developed refeeding hypophosphatemia, nearly 33% of patients developed edema, and low bone mineral density was diagnosed in almost 90% of the patients. Highly elevated LFTs (>3x upper limits of normal) predicted hypoglycemia, and low BMI predicted refeeding hypophosphatemia (*p* = .001).

**Conclusions:**

Although conclusions drawn from the findings presented in this descriptive study must be tempered by relevant clinical judgement, these findings showcase that patients with extreme AN are at significantly increased risk for many serious medical complications secondary to their state of malnutrition and also with initial refeeding.

## Plain English summary

Extreme anorexia nervosa (AN) is defined as a BMI < 15 kg/m^2^ in those meeting DSM-5 diagnostic criteria for AN. However, the frequency of medical complications specific to those meeting this greatest amount of weight loss is unknown. This retrospective study seeks to better define the frequency of medical complications in individuals < 65% ideal body weight admitting to an inpatient medical stabilization unit. We found that patients with extreme AN present with similar medical complications, although at a more frequent occurrence, to those at higher percent ideal body weights. Low bone mineral density is an almost universal finding in this population, with 45% of individuals presenting with osteopenia and 43% with osteoporosis. 35% of individuals developed hypophosphatemia and 38% developed hypoglycemia in this population, both more frequent in the restricting than binge-purge subtype of AN. Elevated liver function tests predict the development of hypoglycemia. Patients with extreme AN are at a significantly increased risk for many serious medical complications secondary to their state of malnutrition and also with initial refeeding.

## Background

Anorexia nervosa (AN) is a serious psychiatric illness that leads to medical complications which can adversely involve every organ system [1]. Mortality remains one of the highest of any psychiatric disorders [2] with cause of death often attributed to the medical complications of malnutrition [3, 4]. The prognosis for AN is somewhat guarded, with less than one-third of individuals recovered at 9 years and approximately two-thirds of individuals recovered when followed up to 22 years [5]. Correlational data suggest that full weight restoration is a predictor of a more favorable outcome at follow up [6, 7], but the lower the percentage of ideal body weight (%IBW) at presentation, the worse the prognosis due to more medical complications [8]. These patients may often undergo unnecessary evaluations for their medical complaints and languish in medical hospitals without definitive care.

The ACUTE Center for Eating Disorders at Denver Health (ACUTE), established in 2004, is a telemetry medical stabilization unit, with national and international draw, that singularly specializes in the care of the most medically compromised patients with eating disorders, as the typical patient admitted to ACUTE meets criteria for an extreme form of the illness per the DSM-V severity index for eating disorders [9]. In this article we present data from a series of 281 patients admitted to ACUTE, as the first study of a comprehensive evaluation of medical complications observed in patients with extreme AN.

## Methods

Patients admitted to ACUTE are regularly transferred by air ambulance from medical hospitals where they had been admitted with various serious medical conditions such as syncope, cardiac arrhythmias, hypoglycemic seizures, failure to thrive, severe electrolyte imbalances and liver failure. Typical ACUTE patients have AN, restricting subtype (AN-R) or binge-purge subtype (AN-BP), with body weights less than 70% of ideal body weight (IBW), and with comorbid medical complications and marked physical deconditioning. Percent IBW is calculated using the Hamwi method [10]. Multidisciplinary expertise, in the medical complications of AN and refeeding, is exclusively provided by embedded full-time internal medicine physicians, psychiatrists, registered dietitians, clinical psychologists, physical therapists, occupational therapists, social workers, nurses, and certified nursing assistants. Standard blood tests, anthropometrics, 12-lead electrocardiograms (EKG), and vital signs with orthostatic blood pressures are obtained for all patients upon admission. Laboratory values, such as glucose, phosphorus, and liver function tests (LFTs) are closely monitored until stabilized. Daily blinded weights were also obtained at approximately the same time daily for each patient while wearing only a gown. The multidisciplinary team then facilitates the initiation of aggressive, albeit judicious, nutritional rehabilitation. Progressive oral (PO) kcals, increased until meeting our unit’s expected weight trends of 0.2 kg/day, is provided with conversion to enteral nutrition (EN) only when patients are unable to tolerate PO intake; rarely, peripheral parenteral nutrition/total parental nutrition (PPN/TPN) is provided when unable to tolerate enteral feeds for significant physiologic reasons. In addition, all meal completion is monitored by a care patient safety aide with training in eating disorders. Once the patient has met ACUTE’s discharge criteria, as well as meeting the accepting treatment program’s admission criteria, they are discharged.

This retrospective study design involves 281 male and female adult patients who weighed less than 65% of IBW and admitted to ACUTE for medical stabilization, between May 18, 2013 and August 30, 2018. It compares baseline characteristics and medical complications between restricting and binge-purge subtypes. Patients were excluded if they were hospitalized for less than 72 h or if their percent IBW was above 65% on admission.

Patient demographics, anthropometric measurements, dietary intake, bone mineral density (BMD) tests, and EKG data were obtained from manual chart review. Nursing assessments and laboratory testing results were queried from the hospital database. Duration of illness and mode and frequency of purging behaviors were self-reported and recorded on admission. Hypothermia was defined as body temperature less than 36 °C and bradycardia was defined as heart rate less than 60 beats per minute (bpm). Severe hypoglycemia was defined as blood glucose < 2.2 mmol/L. Hypophosphatemia was defined as a serum value less than 0.87 mmol/L. Patients with hypophosphatemia on admission were excluded from the risk factor analysis for refeeding hypophosphatemia. Dual-energy X-ray absorptiometry (DXA) scans and lipodystrophy analysis/whole body scans were performed using the Hologic QDR Series model Discovery-W if not already completed within the previous 2 years. BMD was assessed using the lowest regional (hip, femoral neck, and lumbar spine) Z-score or T-score (if patient was 50 years old or older). On ACUTE, osteoporosis is diagnosed if the Z or T-score is less than − 2.5 and osteopenia is diagnosed when the lowest Z or T-score is between − 1 and − 2.5. Nadir body mass index (BMI) and percent IBW are listed to reflect the lowest body weight experienced during treatment. Admission and discharge caloric intake were based on those amounts consumed on the first and last day the patient was on the unit for 24 h. The study was reviewed and approved by the Colorado Multiple Institutional Review Board.

### Statistical analysis

Univariate statistics were used to describe the cohort. Shapiro-Wilks test was used to determine the distribution of continuous variables. Depending on the distribution, patient characteristics, used to describe the medical complications on the ACUTE unit, were ascertained by Mann-Whitney *U* procedures, Pearson chi-square tests, and independent sample *t*-tests. When appropriate, degrees of freedom are shown in parentheses after the test statistic. Logistic regression analyses were used to investigate associations between medical complications with clinical or biochemical variables. Two-tailed *p* values < 0.05 were considered statistically significant, and all analyses were completed using SAS Enterprise Guide software version 7.1 (SAS Institute, Cary, NC).

## Results

### Baseline characteristics

Demographic characteristics and statistical comparisons between AN subtypes, in regard to percent IBW, BMI, and caloric intake, are listed in Table 1. A large majority of ACUTE patients (91%) were referrals from outside of Colorado. Most of the 281 study patients were female (91%), 62% were diagnosed as AN-R subtype, and the age range of the patients was 18–66 years with a median age of 28 years. The average BMI on admission was 12.1 kg/m^2^ (SD = 1.3; range = 7.5–15.7) and average percent IBW was 57.7% (SD = 5.5; range = 34.8–65.0). The median duration of illness was 10 years (IQR = 5–20). For females, duration of illness was significantly higher with a median of 11 years (IQR = 6–21) and for males, the median was 4 years (IQR = 1–10), *p* < .0001. Out of the total cohort, 82 (29%) patients reported vomiting as a method of purging, 41 (15%) reported using laxatives, and 7 (2%) reported using diuretics. Many of these patients used just one method of purging, but some used two or even all three of the methods. However, many patients were unable to recall the frequency of their purging behaviors.

**Table 1:** Characteristics of study participants

	Cohort (***N*** = 281)		AN-R (***N*** = 175)		AN-BP (***N*** = 106)			
Patient Characteristics	N	%	N	%	N	%		
Female	256	91	158	62	98	38		
Male	25	9	17	68	8	32		
**Admitted from**								
Home	219	78	128	58	91	42		
Hospital transfer	37	13	29	78	8	22		
Inpatient eating disorder program	23	8	17	74	6	26		
Other	2	< 1	1	50	1	50		
Referral from outside Colorado	257	91	161	63	96	37		
	**Mean**^**a**^	**SD**^**a**^	**Mean**^**a**^	**SD**^**a**^	**Mean**^**a**^	**SD**^**a**^	***t*-test**^**b**^	***p*value**
Age	28	22–37	27	22–38	31	22–37	15,900	0.15
Length of hospitalization (days)	22	15–30	23	16–33	21	13–28	13,754	0.07
**Percent of IBW**								
Admission	57.7	5.5	57.2	5.5	58.4	5.4	1.75	0.08
Nadir	57	6.2	56.5	6.1	57.9	6.5	1.81	0.07
Discharge	69	7.4	67.7	6.2	71.2	8.7	3.61	< .001
Change in %IBW	12	8.4	11.2	7.5	13.3	9.6	2.03	0.04
**BMI (kg/m**^**2**^**)**								
Admission	12.1	1.3	12	1.3	12.2	1.3	1.01	0.31
Nadir	12	1.4	11.9	1.4	12.1	1.6	0.91	0.37
Discharge	14.5	1.5	14.2	1.3	14.9	1.7	3.5	< .001
Change in BMI	2.5	1.5	2.3	1.4	2.8	1.7	2.8	<.001
Weekly weight gain (kg)	2	1.5	1.7	0.9	2.6	2.0	4.26	< .0001
**Daily caloric intake (kcal)**								
Admission	1457	249	1477	269	1424	209	−1.74	0.06
Discharge	3175	762	3282	763	2996	728	−3.1	0.002

*AN-R* Anorexia nervosa restricting subtype, *AN-BP* Anorexia nervosa binge-purge subtype, *IBW* Ideal body weight, *BMI* Body mass index

^a^Median and IQR for not normally distributed variables

^b^Mann-Whitney *U* test

### Patient care and discharge

Patients hospitalized at ACUTE had a median length of stay of 22 days (IQR = 15–30). The average percent IBW increased by 12% (SD = 8.4), and average BMI increased by 2.5 kg/m^2^ (SD = 1.5) by time of discharge (Table 1). Patients with AN-BP gained 0.9 more kilograms per week compared to patients with AN-R (*p* < .0001). About one third of the patients developed edema during their hospital stay and half of those patients, almost entirely patients with AN-BP, were prescribed spironolactone to treat their excessive weight trends and/or edema. Patients with AN-BP, who were prescribed spironolactone, gained 0.7 kg more per week than patients with AN-BP who were not prescribed spironolactone.

Most of the patients discharged voluntarily (*n* = 209) with an average percent IBW of 69.4% (SD = 7.6); however, 37 patients discharged from ACUTE on a mental health hold (MHH), 21 discharged on a short-term certification (STC), and 14 discharged against medical advice (AMA).

All patients were medically stabilized prior to discharge. Two hundred forty-one patients (86%) discharged to an inpatient or residential program; however, 16 patients (6%) discharged to a partial hospitalization program (PHP), an outpatient team, or to another form of care, and the remaining 24 patients (9%) were discharged home due to mitigating circumstances. No patient died on the ACUTE unit during their medical stabilization and only 20 patients (7%) ever required brief transfer to the Denver Health medical intensive care unit (ICU) for conditions such as critical hyponatremia (< 116 mmol/L), critical hypokalemia (< 1.8 mmol/L), refractory hypoglycemia requiring intravenous (IV) glucose and frequent point of care glucose monitoring, small bowel obstructions requiring surgery, cardiogenic shock, upper gastrointestinal bleed requiring transfusion, and two with sustained ventricular tachycardia. Forty-three patients (15%) were deemed high fall risk upon admission, and 15 patients (5%) were initially incapable of ambulation. All received intensive physical therapy and were able to ambulate on their own at the time of discharge.

### Nutrition

On admission, initial caloric intake for females ranged from 800 to 2450 kcals with an average of 1431 kcal/day (SD = 191) and initial caloric intake for males was significantly higher at 1714 kcal/day (SD = 507) (*p* = .01). The vast majority of patients were refed via progressive oral nutrition. There were 50 patients (18%) who required enteral feeds; 18 patients (6%) required some parenteral nutrition. Average daily caloric intake at the time of discharge for females was 3119 kcal/day (SD = 733) and 3744 kcal/day for males (SD = 829). During their ACUTE stay, patients gained 2 kg/wk. (SD = 1.5). There was not a significant difference in weight gain per week between genders (*p* = .84).

### Medical findings

Patients were hypothermic during 35% of their total admission time at ACUTE with low percent IBW as a predictor of hypothermia (*p* < .0001). For patients with AN-R, the average percent IBW on the last hospital day of hypothermia was 61.5% (SD = 6.3) (Table 2).

**Table 2:** Medical complications between AN-R and AN-BP subtypes

	Cohort (***N*** = 281)		AN-R (***N*** = 175)				AN-BP (***N*** = 106)					
	N	%	N	%	Mean^**a**^	SD^**a**^	N	%	Mean^**a**^	SD^**a**^	***t***-test^**b**^	***p*** value
**Body Temperature**												
Hypothermic during stay (< 36 °C)	168	60	110	65	35.8	0.1	58	35	35.8	0.2	1.59	0.11
Last hospital day < 36 °C					10	4–19			11	5–21	6084	0.85
%IBW on last day < 36 °C					61.5	6.3			65.8	7.2	4.12	< .0001
**Heart Rate and EKG on Admission**												
Heart rate (bpm)					55.3	13.1			60.7	12.5	3.37	0.0009
Nadir heart rate (bpm) during stay					50.1	11.8			54.3	3.9	3.06	0.002
QTc value (ms)					421	31.4			436	37.2	1.41	< .001
High QTc (480–500 ms)	7	3	2	29			5	71				
Critically high QTc (> 500 ms)	7	3	3	43			4	57				
Junctional rhythm	5	2	1	20			4	80				
Right axis	58	21	40	69			18	31				
**Liver Function**												
All normal LFTs (AST < 41; ALT < 46 U/L)	85	30	36	42			49	58				
Modestly Abnormal (up to 3x normal)	101	36	60	59			41	41				
Severely abnormal (> 3x normal)	95	34	79	83			16	17				
Highest AST					197	145–348			238	136–537	383	0.76
Highest ALT					270	197–485			261	196–518	618	0.65
Hospital day LFTs peaked					4	0–12			5	0–12	6381	0.21
**Blood Glucose**												
Hypoglycemia (< 3.3 mmol/L)	107	38	77	72	3.0	2.6–3.2	30	28	2.9	2.8–3.1	1637	0.45
Last hospital day glucose < 3.3 mmol/L					2	1–2			1	0–2	1963	0.06
Severe hypoglycemia (< 2.2 mmol/L)^c^	24	9	20	83	1.8	1.6–2.0	4	17	1.9	1.4–2.1		
Last hospital day glucose < 2.2 mmol/L					1	0–3			1	0–3		

*AN-R* Anorexia nervosa restricting subtype, *AN-BP* Anorexia nervosa binge-purge subtype, *IBW* Ideal body weight, *EKG* Electrocardiogram, *bpm* Beats per minute, *LFT* Liver function tests, *AST* Aspartate aminotransferase test, *ALT* Alanine aminotransferase test

^a^Median (IQR) for not normally distributed variables

^b^Mann-Whitney *U* test

^c^There were too few values for an analysis between AN subtypes and severe hypoglycemia

There were 158 patients (56%) who were bradycardic on arrival to ACUTE, with the lowest recorded heart rate being 26 bpm, and the majority of those patients were diagnosed with AN-R. Admission BMI (*r* = .85, *p* < .0001) and percent IBW (*r* = .85, *p* < .0001) were highly correlated with bradycardia. For the patients with bradycardia, their heart rate had somewhat improved by median day 4 (IQR = 1–9). Findings from the admission EKG revealed that the average QTc was normal at 427 ms (SD = 34.4). Patients with AN-BP had significantly longer average admission QTc (436 ms, SD = 37.2) than patients with AN-R (421 ms, SD = 31.4), (*p* < .001).

Cardiac axis was mostly normal. There were 58 patients with right axis deviation but only 10 patients presented with left axis deviation. Five patients presented with a junctional rhythm. Admission BMI was not a significant predictor of either right axis deviation (*p* = .45) or of developing a junctional rhythm (*p* = .15).

Just over half of the patients admitted with low serum prealbumin values (< 0.2 g/L). Low serum prealbumin positively correlated with admission percent IBW (*r* = 0.6, *p* < .0001). Admission serum albumin and alkaline phosphatase levels were within normal ranges. However, 127 (45%) patients had elevated admission aspartate aminotransferase (AST) values (above 40 U/L) and 145 (52%) patients had elevated alanine aminotransferase (ALT) values (above 45 U/L). Patients with AN-R had significantly higher AST (*p* < .0001) and ALT (*p* < .0001) values compared to patients with AN-BP (Table 3).

**Table 3:** Admission laboratory values between AN subtypes (*N* = 281)^a^

	AN-R		AN-BP		***t***-test^**b**^	***p*** value
Laboratory Values (reference range)	Mean^**c**^	SD^**c**^	Mean^**c**^	SD^**c**^		
Sodium (135–143 mmol/L)	137.6	4.5	135	6.4	−4.01	<.0001
Potassium (3.6–5.1 mmol/L)	3.8	0.4	3.4	0.7	−6.39	<.0001
Carbon Dioxide (18–27 mmol/L)	27.7	3.3	29.3	7.3	2.57	0.01
Glucose (3.3–11.0 mmol/L)	4.57	3.91–5.89	5.26	4.29–6.0	16,507	0.009
Calcium (2.02–2.6 mmol/L)	2.1	0.1	2.2	0.2	5.11	<.001
Phosphorus (0.87–1.58 mmol/L)	1.03	0.2	1.1	0.4	1.69	0.09
Magnesium (0.65–1.1 mmol/L)	0.95	0.1	0.98	0.2	1.60	0.11
BUN (0.21–0.79 mmol/L)	0.57	0.36–0.86	0.50	0.32–0.75	13,987	0.07
Creatinine (44.2–122.9 μmol/L)	59.3	19.1	73.9	28.6	4.65	<.0001
AST (10–40 U/L)	49	26–113	27	21–42	11,730	<.0001
ALT (7–45 U/L)	73	37–194	32	24–50	10,671	<.0001
Total bilirubin (17.1–20.5 μmol/L)	8.6	6.8–12.0	6.8	5.1–15.4	14,294	0.16
Albumin (30–53 g/L)	38.5	4.9	37.9	5.9	−0.85	0.39
Alkaline phosphatase (35–137 U/L)	67	51–97	68	54–95	15,213	0.68
Total protein (60–82 g/L)	67.0	7.5	68.7	9.1	1.78	0.08
WBC (4.5–10.0 10^9^ L^−1^)	3.8	1.6	5.5	3.3	5.91	<.0001
Neutrophils (%) (48.0–69.0%)	56.9	12.6	57.4	14.5	0.27	0.79
Lymphocytes (%) (21.0–43.0%)	34.4	12.4	33.3	13.5	−0.71	0.48
Platelet count (150–400 10^9^ L^−1^)	192	142–250	271	201–387	19,308	<.0001
Hematocrit (%) (37.0–47.0%)	36.6	5.8	37.6	5.6	1.4	0.16
MCV (80–100 fL)	94.6	6.7	91.1	6.7	−4.27	<.0001
TSH (0.34–6.0 mU/L)	2	1.2–3.1	1.4	1–2.2	11,935	<.001
INR (0.83–1.19)	1.2	0.2	1.1	0.2	−3.49	<.001
1,25 Hydroxy vitamin D (74.9–199.7 nmol/L)	97.3	77.4–128.5	97.3	64.9–132.3	13,641	0.19
Testosterone (5.6–25.2 nmol/L)	5.9	2.1–11.7	5.9	4.4–14.6	74.0	0.39
Free T3 (4.0–7.4 pmol/L)^d^	3.1	2.0–3.7	2.3			
Free T4 (6.4–24.5 nmol/L)	11.1	9.1–12.9	13.1	11.8–16.3	632.0	0.01
Prealbumin (0.2–0.52 g/L)	0.18	0.06	0.21	0.07	3.61	<.001

*AN-R* Anorexia nervosa restricting subtype, *AN-BP* Anorexia nervosa binge-purge subtype, *BUN* Blood urea nitrogen, *AST* Aspartate aminotransferase test, *ALT* Alanine aminotransferase test, *WBC* White blood cell, *MCV* Mean corpuscular volume, *TSH* Thyroid stimulating hormone, *INR* International normalized ratio, *Free T3* Triiodothyronine, *Free T4* Thyroxine

^a^Magnesium and TSH were available on 274 admissions. Neutrophils, lymphocytes, and 1,25 Hydroxy Vitamin D were available on 275 admissions. INR was available on 280 admissions. Testosterone was available on 19 admissions. Free T3 was available on 7 admissions. Free T4 was available on 55 admissions. Prealbumin was available on 277 admissions

^b^Mann-Whitney *U* test

^c^ Median (IQR) for not normally distributed variables

^d^There were too few values for an analysis between AN subtypes and Free T3 (*n* = 7)

The incidence of increased liver function tests (LFTs), on admission, is correlated with low BMI [χ^2^(1) = 13.3, *p* < .005]. Of the 281 study patients, only 85 (30%) had normal LFTs (AST < 41 U/L and ALT < 46 U/L) throughout their hospitalization. One hundred one (36%) had modestly elevated LFTs and 95 patients (34%) had LFTs of greater than three times normal during their hospitalization (Table 2). Modestly abnormal LFTs predicted thrombocytopenia (serum platelet count < 150 10^9^ L^− 1^) on admission (*p* = .002) but did not predict hypoglycemia (*p* = 0.43). However, highly elevated LFTs (>3X normal) did predict the presence of hypoglycemia (*p* = .005).

More than one third (38%) of the patients experienced hypoglycemia while on ACUTE. Of these, 24 (9%) admitted with severe hypoglycemia (< 2.2 mmol/L.). Of the total 281 patients, 77 patients (27%) with AN-R developed hypoglycemia and 20 patients developed severe hypoglycemia. There were only 30 patients (11%) with AN-BP who developed hypoglycemia and only four developed severe hypoglycemia (Table 2).

Both admission sodium and potassium values, were lower in patients with AN-BP compared to patients with AN-R (M = 135 mmol/L, SD = 6.4 vs. M = 137.6 mmol/L, SD = 4.5, *p* < .0001). Hypokalemia was most commonly treated with a combination of oral potassium and IV fluids (47%), only oral potassium (28%), oral and IV potassium along with IV fluids (13%), combination oral and IV potassium (9%), or only IV fluids (3%). Only eight patients of those developing hypokalemia (*n* = 93) required transfer to the ICU for critically low values. Hyponatremia was most commonly treated with slow IV saline (50%), 34% were allowed to auto-correct without any particular intervention aside from nutrition and fluid intake, and 13% required fluid restriction due to a diagnosis of syndrome of inappropriate antidiuretic hormone (SIADH). Four (5%) of the total 82 patients, who developed hyponatremia, required transfer to the ICU for treatment of a critical value.

There were 179 patients (64%) who admitted with low white blood cell counts (leukopenia), 47% with anemia and 20% with thrombocytopenia. Leukopenia and thrombocytopenia were more marked in patients with AN-R versus AN-BP (M = 3.8 10^9^ L^− 1^, SD = 1.6 vs. M = 5.5 10^9^ L^− 1^, SD = 3.3, *p <* .0001) and (Mdn = 192 10^9^ L^− 1^, IQR = 142–250 vs. Mdn = 271 10^9^ L^− 1^, IQR = 210–387, *p* < .0001), respectively (Table 3).

There were 60 patients on admission with a serum phosphorous level < 0.87 mmol/L and average %IBW was 55.3% (SD = 6.4). An additional 35% (5 males and 93 females) developed refeeding hypophosphatemia, as early as median day 2 (IQR = 1–2). Of those patients, 47% (3 males and 43 females) developed critical hypophosphatemia (serum phosphorus < 0.71 mmol/L). 49% of patients with critical hypophosphatemia required IV phosphorus for an average of 1.6 days (SD = 0.8), and all patients developing hypophosphatemia were treated for an average of 6.1 days (SD = 5.1) of oral phosphorous repletion. Patients maintained low serum phosphorous values < 0.87 mmol/L for a mean of 2.1 (SD = 1.3) days with use of supplement. When comparing the 98 patients who developed refeeding hypophosphatemia, with the 123 patients who never developed refeeding hypophosphatemia, there were more patients diagnosed with AN-R than patients diagnosed with AN-BP [23% vs. 21%, χ^2^(1) = 5.44, *p* = .02], but there were no significant differences between gender (*p* = .14). However, there was a significant difference in average nadir BMI between patients who developed refeeding hypophosphatemia (M = 11.8 kg/m^2^, SD = 1.4) during their hospitalization compared to patients who did not (M = 12.4 kg/m^2^, SD = 1.3, *p* = .001) (Table 4).

**Table 4:** Predictors of refeeding hypophosphatemia^a^

	Hypophosphatemia (***N*** = 98)		Normal Phosphorus (***N*** = 123)			
Factors	Mean^**b**^	SD^**b**^	Mean^**b**^	SD^**b**^	***t-***test^**c**^	***p*** value
Nadir BMI	11.8	1.4	12.4	1.3	3.31	0.001
Nadir %IBW	56.7	6.3	58.5	5.5	2.21	0.03
Admission Glucose (mmol/L)	4.7	3.9–5.6	4.4	3.9–5.2	11,630	0.06
Admission Albumin (g/L)	36.8	6.8	38.0	6.3	− 1.41	0.16
Admission Prealbumin (g/L)	0.19	0.08	0.21	0.06	−1.87	0.06
Admission WBC (10^9^ L^− 1^)	4.8	2.5	4.3	2.7	−1.41	0.16
Lean Mass (g)^d^	27,597	4351	28,578	5702	−0.92	0.36
	**N**	**%**	**N**	**%**	**χ2**	***p*value**
Elevated admission AST (> 40 U/L)	41	42	45	37	0.63	0.42
Elevated admission ALT (> 45 U/L)	49	50	54	44	0.81	0.37

*BMI* Body mass index, *IBW* Ideal body weight, *WBC* White blood cell, *AST* Aspartate aminotransferase test, *ALT* Alanine aminotransferase test

^a^Hypophosphatemia defined as phosphorus < 0.87 mmol/L. 60 patients with hypophosphatemia on admission were excluded

^b^Median (IQR) for not normally distributed variables

^c^Mann-Whitney *U* test

^d^Data for lean mass (g) was available for 38 patients with refeeding hypophosphatemia and 66 patients with normal phosphorus

One hundred thirty-nine patients (49%) received DXA scans during their hospitalization. Only 12% had normal BMD (4% male), 45% osteopenia (6% male), and 43% osteoporosis (5% male). The average Z-score in males was − 1.8 (SD = 1.3) and in females, − 2.3 (SD = 1.2). There were no significant differences when comparing Z-scores between patients with AN-R and AN-BP (*p* = .24) (Table 5). The average T-score for males was − 3.9 (SD = 0.9), which was much worse than the average T-score for older females (M = -2.6, SD = 1.0).

**Table 5:** Bone mineral density and lipodystrophy studies between AN subtypes

	Cohort (***N*** = 139)	AN-R (***N*** = 94)		AN-BP (***N*** = 45)			
DXA scans		N	%	N	%		
Normal BMD (Z/T-score > −1)	17	11	65	6	35		
Osteopenia (Z/T-score − 1 to −2.5)	62	45	73	17	27		
Osteoporosis (Z/T-score < − 2.5)	60	38	63	22	37		
		**Mean**	**SD**	**Mean**	**SD**	***t*-test**	***p*value**
Z-score	127	−2.2	1.2	−2.4	1.1	−1.17	0.24
T-score^a^	12	−2.7	1.2	−3.7	0.4		
**Lipodystrophy studies**							
		**Median**	**IQR**	**Median**	**IQR**	**Mann-Whitney** ***U*test**	***p*value**
Body Fat (g)	134	3799	3398-4709	4329	3566-5317	3408	0.04
Body Fat (%)	134	11.6	10.8–13.4	12.8	11.2–16.1	3486	0.01
Lean Mass (%)	134	77.8	75.2–80.0	76.4	74.4–78.1	2465	0.02

*AN-R* Anorexia nervosa restricting subtype, *AN-BP* Anorexia nervosa binge-purge subtype, *DXA* Dual-energy X-ray absorptiometry, *BMD* Bone mineral density, *IQR* Interquartile range

^a^There were too few values for an analysis between AN subtypes and T-scores (*n* = 12)

## Discussion

This is the largest study ever of severely malnourished adult patients with extreme AN, with percent IBW less than 65%, hospitalized on a medical stabilization unit. In comparison to our much smaller pilot study, AN patients are now more ill, manifested by an average admission BMI of 12.1 kg/m^2^ (range = 7.5–15.7) compared to our previous 13.1 kg/m^2^ (range = 9.5–15.4). Length of stay has increased from 19 to 22 days, but discharge BMI remained similar (14.5 kg/m^2^ vs 14.4 kg/m^2^). We have also become much more aggressive in our refeeding practices, with our admission kcals increasing from 990 to 1400 and discharge kcals increasing from 2000 kcal to approximately 3000 kcal [11].

When analyzed by type of eating disorder, patients with AN-R gained less weight per week than patients with AN-BP. We do not, however, believe that patients with AN-BP inherently gain weight faster than patients with AN-R, as previously suggested [12]. In that study, there was no mention of edema formation in the AN-BP group, nor how often the aldosterone antagonist spironolactone was utilized. Excessive purging behaviors lead to a secondary hyperaldosteronism (Pseudo Bartter’s syndrome) which makes these patients salt and water retentive, resulting in excessive weight trends and/or the development of edema [13, 14]. In our study, 89% of patients with AN-BP were treated with spironolactone for an average of 13 days.

Curiously, only 56% of the patients in this study were documented as having bradycardia, which is considered a universal finding in AN, especially in those patients with lower levels of body weight. Indeed, this study corroborated the correlation between bradycardia and percent IBW. Given this is a retrospective study, heart rates were obtained from the nurses’ documented values, which are only obtained intermittently throughout the day; however, if 24-h telemetry values were instead used, it is likely the frequency of bradycardia would approach 100% especially with the known further lowering of heart rate at night.

Patients with AN-BP had a longer QTc interval than patients with AN-R, likely due to electrolyte imbalance. Indeed, patients with AN-BP had lower admission potassium levels which are known to prolong the QTc. Importantly, once again, no correlation was found between QTc and admission BMI thus reinforcing the fact that a prolonged QTc is not inherent to AN. Rather, when detected, a prolonged QTc is a consequence of electrolyte disturbances or the usage of QT-prolonging medications [15–17].

In our cohort, 70% of patients had abnormal LFTs which peaked on the fourth hospital day for patients with AN-R and day five for patients with AN-BP. On average, the ALT was greater than the AST across all subtypes of eating disorders with AN-R patients having higher transaminases than AN-BP patients. Elevations in transaminases are the most common kind of liver abnormality seen in patients with severe AN [18, 19]; bilirubin and alkaline phosphatase levels are almost never elevated. In our cohort, 40% of patients with a BMI < 12.1 kg/m^2^ had elevated transaminases, thus supporting the relationship between severity of AN and likelihood of transaminitis. The most likely pathophysiologic mechanism is starvation-mediated autophagy [20, 21].

Hypoglycemia was more likely to develop in those with elevation of transaminases greater than three times normal and in those with greater severity of malnutrition, as evidenced by 27% of those with AN-R developing hypoglycemia vs. 11% of individuals with AN-BP. Thus, hypoglycemia as a surrogate of severity of illness was supported [22, 23]. This is very important because our experience suggests lean people do not typically have the customary adrenergic or neuroglycopenic warning symptoms typical of severe hypoglycemia [22].

Serum albumin levels were notably within the normal range, notwithstanding their marked malnutrition. However, more than 50% of our patients admitted with a low prealbumin level < 0.2 g/L, with AN-R patients having the lowest values. These findings are supported by an emerging literature that albumin is not considered a reliable indicator of the severity of AN [23, 24]. Rather, albumin is best viewed as an acute phase reactant that decreases predictably in critical illness such as sepsis [25]. The precise cause of low serum prealbumin levels in severe AN remains to be elucidated [24, 26]. Furthermore, low serum prealbumin is associated with both refeeding hypophosphatemia [27] as well as hypoglycemia in AN.

Patients with AN-R had more pronounced leukopenia and thrombocytopenia than those with AN-BP. In severe AN, the bone marrow shows signs of serous fat atrophy [28–30], which is directly related to duration of illness and BMI [31]. This corrects with a judicious nutritional plan.

The most common electrolyte abnormality on admission, in our cohort, was hypokalemia, with 33% of patients having a serum potassium level <  3.6 mmol/L, followed by hyponatremia. Hypernatremia was very rare. When examined by eating disorder sub-type, patients with AN-BP had, not surprisingly, statistically significant lower sodium and potassium levels on admission. The hyperaldosteronism resulting from the dehydration of purging along with loss of potassium and other electrolytes in emesis, stool, and urine contribute to hypokalemia [32, 33]. The ability to correct hypokalemia is improved with down-regulation of aldosterone secretion, which can be accomplished with intravascular volume repletion; hence the large number of patients that received IV fluids in the treatment of their hypokalemia.

Notably, only 21% of this cohort admitted with hypophosphatemia, but over one third of our patients subsequently developed refeeding hypophosphatemia by hospital day two. Compared to patients who did not develop hypophosphatemia, those who did, had lower admission BMI, thus representing a sicker patient population who is at greater risk for refeeding hypophosphatemia [34]. Studies have indeed shown that as BMI nears 12 kg/m^2^, medical complications such as refeeding hypophosphatemia occur more frequently [27, 35, 36]. Based on this present study, patients who manifested a tendency towards developing refeeding hypophosphatemia had lower BMIs, higher transaminases, and lower serum prealbumin levels. Caloric intake during admission, per se, did not predict hypophosphatemia. However, notwithstanding the extremely ill population of patients cared for on ACUTE, 44% never developed refeeding hypophosphatemia. We therefore feel it unnecessary to prophylactically give phosphorus replacement to all patients at the time of admission given the possibility that it can cause diarrhea.

Lastly, 139 of our patients received a DXA scan during their admission and of those, 88% were found to have BMD loss [osteopenia (45%) and osteoporosis (43%)]. Although prior studies have shown rates of osteoporosis in males to be worse than females [37], in our current study, females had overall higher rates of osteoporosis (43% vs 5%). Weight restoration is the gold standard for treatment of osteoporosis [38, 39]. Further studies are needed to elucidate which available pharmaceutical options may also be valuable for this patient population [40].

While the strength of this article is that it describes the medical complications in the largest ever published cohort of male and female patients with extreme AN, there are limitations. First, it was based on retrospective data review, thereby somewhat limiting the conclusions that can be drawn from this descriptive study. Secondly, we included patients transferred from other hospitals as well as other eating disorder treatment facilities and we cannot account for the care and refeeding protocols the patients received there. Third, our sample size remains relatively small as our program focuses exclusively on the care of the extreme form of AN and may reduce the generalizability of our findings. Lastly, we did not include readmissions in this sample size.

In conclusion, this article methodically examines the many medical complications of patients with extreme AN-R and AN-BP in an effort to inform care providers of the multitude of medical issues which may be present in these patients with extreme forms of the illness.

## Data Availability

The datasets generated and analyzed during the current study are available from the corresponding author on reasonable request.

## Abbreviations

ACUTE: ACUTE Center for Eating Disorders at Denver Health
ALT: Alanine aminotransferase
AMA: Against medical advice
AN: Anorexia nervosa
AN-BP: Binge-purge subtype
AN-R: Restricting subtype
AST: Aspartate aminotransferase
BMD: Bone mineral density
BMI: Body mass index
BPM: Beats per minute
DSM: Diagnostic and Statistical Manual of Mental Disorders
DXA: Dual-energy X-ray absorptiometry
EKG: Electrocardiogram
IBW: Ideal body weight
ICU: Intensive care unit
IQR: Interquartile range
IV: Intravenous
LFT: Liver function test
M: Mean
MDN: Median
MHH: Mental health hold
PHP: Partial hospitalization program
SAS: Statistical analysis software
SD: Standard deviation
SIADH: Syndrome of inappropriate antidiuretic hormone
STC: Short-term certification
